# Clinical profile and outcome of surgical treatment of perforated peptic ulcers in Northwestern Tanzania: A tertiary hospital experience

**DOI:** 10.1186/1749-7922-6-31

**Published:** 2011-08-26

**Authors:** Phillipo L Chalya, Joseph B Mabula, Mheta Koy, Mabula D Mchembe, Hyasinta M Jaka, Rodrick Kabangila, Alphonce B Chandika, Japhet M Gilyoma

**Affiliations:** 1 Department of Surgery, Weill-Bugando University College of Health Sciences, Mwanza, Tanzania; 2 Department of Internal Medicine, Weill-Bugando University College of Health Sciences, Mwanza, Tanzania; 3 Department of Surgery, Muhimbili University of Health and Allied Sciences, Dar Es Salaam, Tanzania

**Keywords:** Perforated peptic ulcers, clinical profile, surgical management, outcome, Tanzania

## Abstract

**Background:**

Perforated peptic ulcer is a serious complication of peptic ulcers with potential risk of grave complications. There is paucity of published reports on perforated peptic ulcer disease in our local environment. This study was conducted to evaluate the clinical presentation, management and outcome of patients with peptic ulcer perforation in our setting and to identify predictors of outcome of these patients.

**Methods:**

This was a combined retrospective and prospective study of patients who were operated for perforated peptic ulcers at Bugando Medical Centre between April 2006 and March 2011. Data were collected using a pre-tested and coded questionnaire and analyzed using SPSS computer software version 15.0. Ethical approval to conduct the study was obtained from relevant authority before the commencement of the study.

**Results:**

A total of 84 patients were studied. Males outnumbered females by a ratio of 1.3: 1. Their median age was 28 years and the modal age group was 21-30 years. The median duration of illness was 5.8 days. The majority of patients (69.0%) had no previous history of treatment for peptic ulcer disease. The use of non-steroidal anti-inflammatory drugs, alcohol and smoking was reported in 10.7%, 85.7% and 64.3% respectively. Eight (9.5%) patients were HIV positive with a median CD4 count of 220 cells/μl. Most perforations were located on the duodenum {90.4%) with the duodenal to gastric ulcers ratio of 12.7: 1. Graham's omental patch (Graham's omentopexy) of the perforations was performed in 83.3% of cases. Complication and mortality rates were 29.8% and 10.7% respectively. The factors significantly related to complications were premorbid illness, HIV status, CD 4 count < 200 cells/μl, treatment delay and acute perforation (P < 0.001). Mortality rate was high in patients who had age ≥ 40 years, delayed presentation (>24 hrs), shock at admission (systolic BP < 90 mmHg), HIV positivity, low CD4 count (<200 cells/μl), gastric ulcers, concomitant diseases and presence of complications (P < 0.001). The median overall length of hospital stay was 14 days. Excellent results using Visick's grading system were obtained in 82.6% of surviving patients.

**Conclusion:**

Perforation of peptic ulcer remains a frequent clinical problem in our environment predominantly affecting young males not known to suffer from PUD. Simple closure with omental patch followed by *Helicobacter pylori *eradication was effective with excellent results in majority of survivors despite patients' late presentation in our center.

## Background

Peptic ulcer disease (PUD) represents a worldwide health problem because of its high morbidity, mortality and economic loss [1]. In the United States, approximately 5 million adults suffer annually from peptic ulcer disease and 500.000 new cases with 4 million recurrences are reported each year [1,2].

Globally, the incidence of peptic ulcer disease has fallen in recent years [3-5]. Despite this and recent advances in both diagnosis and management of peptic ulcer disease, namely the improvement in endoscopic facilities, eradication of H. pylori and the introduction of the proton pump inhibitors, complications such as peptic ulcer perforation remain a substantial healthcare problem. This may be due to an increase in the risk factors for peptic ulcer complications [3,6].

Peptic ulcer perforation is a serious complication which affects almost 2-10% of peptic ulcer patients on the average [7,8]. Peptic ulcer perforation presents with an overall mortality of 10% [9] although some authors report ranges between 1.3% and 20% [10,11]. Being a life threatening complication of peptic ulcer disease, it needs special attention with prompt resuscitation and appropriate surgical management if morbidity and mortality are to be avoided [3,11].

The pattern of perforated PUD has been reported to vary from one geographical area to another depending on the prevailing socio-demographic and environmental factors [12]. In the developing world, the patient population is young with male predominance, patients present late, and there is a strong association with smoking [13]. In the west the patients tend to be elderly and there is a high incidence of ulcerogenic drug ingestion [14].

The diagnosis of perforated PUD poses a diagnostic challenge in most of cases. The spillage of duodenal or gastric contents into peritoneal cavity causing abdominal pain, shock, peritonitis, marked tenderness and decreased liver dullness offers little difficulty in diagnosis of perforations [15].The presence of gas under the diaphragm on plain abdominal erect X-ray is diagnostic in 75% of the cases [16].

Since the first description of surgery for acute perforated peptic ulcer disease, many techniques have been recommended. The recent advances in antiulcer therapy have shown that simple closure of perforation with omental patch followed by eradication of *H. Pylori *is a simple and safe option in many centers and have changed the old trend of truncal vagotomy and drainage procedures [17]. The definitive operation for perforated PUD is performed by few surgeons.

Delay in diagnosis and initiation of surgical treatment of perforated PUD has been reported to be associated with high morbidity and mortality after surgery for perforated PUD [4,17]. Early recognition and prompt surgical treatment of perforated PUD is of paramount importance if morbidity and mortality associated with perforated PUD are to be avoided [4,11]. A successful outcome is obtained by prompt recognition of the diagnosis, aggressive resuscitation and early institution of surgical management.

Little work has been done on the surgical management of perforated peptic ulcer disease in our local environment despite increase in the number of admissions of this condition. The aim of this study was to describe our experience on the surgical management of perforated peptic ulcer disease in our local environment outlining the incidence, clinical presentation, management and outcome of patients with peptic ulcer perforation in our setting and to identify predictors of outcome of these patients.

## Methods

### Study design and setting

This was a combined retrospective and prospective study of patients operated for peptic ulcer perforations at Bugando Medical Centre (BMC) in Northwestern Tanzania from April 2006 to March 2011. BMC is a tertiary care hospital in Mwanza City that also receives patients from its six neighboring regions around Lake Victoria. It is a 1000 bed, consultant and teaching hospital for the Weill-Bugando University Collage of Health Sciences (WBUCHS) and other paramedics.

### Study subject

The subjects of this study included all patients who were operated for perforated peptic ulcers at Bugando Medical Centre during the period under study. Patients with incomplete data were excluded from the study. Patients treated conservatively and those who failed to consent for HIV infection were also excluded from the study. The details of patients who presented from April 2006 to March 2008 were retrieved retrospectively from patient registers kept in the Medical record departments, the surgical wards, and operating theatre. Patients who presented to the A & E department between April 2008 and March 2011 were prospectively enrolled in the study after signing an informed written consent for the study. A detailed history and thorough physical examination were followed by investigations like full blood count, blood grouping, serum urea, serum creatinine and random blood sugar. Patients were also screened for HIV infection using rapid test/ELISA test. A determination of CD 4 count was also performed in all HIV positive patients. Radiological investigations like X-ray abdomen erect and chest X-ray were done in all patients on the suspicion of diagnosis of perforated PUD. Other investigations included hematological profile, serum urea and electrolytes and urinalysis. The diagnosis of perforated PUD was made from history, plain abdominal and chest radiographs, and confirmed at laparotomy. Patients were put on intra-venous fluids, nasogastric suction, intravenous antibiotics and intravenous anti-ulcer drugs; adequate hydration was indicated by an hourly urine output of 30 ml/hour. After adequate resuscitation, laparotomy was done through midline incision and identified the perforation site. Simple closure of the perforation and reinforcement with pedicled omental patch (Graham's omentopexy) was done. Thorough peritoneal lavage with 3 to 4 liters of normal saline was followed by placement of intraperitoneal drain. The operations were performed either by a consultant surgeon or a senior resident under the direct supervision of a consultant surgeon. The Boey score [11] as a tool for outcome prediction was calculated based on data recorded at the time of admission to hospital. The Boey risk stratification in perforated peptic ulcer consists of associated medical illness, preoperative shock and long-standing perforation (more than 24 hours). Preoperative shock was defined as a preoperative systolic blood pressure of less than 90 mmHg. All the patients were put on triple regime consisting of Amoxicillin (500 mg TID), Metranidazole(400 mg TID) and Omeprazole (20 mg BID), all given orally for 14 days to eradicate *H. Pylori. *Patients were followed up on an out patient basis for up to 12 months after surgery. Depending upon their symptoms at each visit, patients were graded using a modified Visick classification [18] as follows:-

Grade I: No symptoms, excellent results.

II: Mild symptoms, good results.

III: Moderate symptoms, easily controlled by medications.

IV: Severe symptoms, requiring constant medication or re-operation

### Data collection

Data were collected using a preformed questionnaire. variables included in the questionnaire were; patient's demographic data (age, sex), associated medical premorbid illness, duration of illness, previous history of PUD, NSAID use, alcohol use and cigarette smoking, HIV status, CD 4 count, timing of surgical treatment, site of perforation, size of perforation, type of surgical procedure, postoperative complication, length of hospital stay, mortality. The duration of symptoms was defined as the time span between the initial pain perception due to perforation and the operation.

### Statistical analysis

The statistical analysis was performed using statistical package for social sciences (SPSS) version 15.0 for Windows (SPSS, Chicago IL, U.S.A).The mean ± standard deviation (SD), median and ranges were calculated for continuous variables whereas proportions and frequency tables were used to summarize categorical variables. Continuous variables were categorized. Chi-square (χ2) test were used to test for the significance of association between the independent (predictor) and dependent (outcome) variables in the categorical variables. The level of significance was considered as P < 0.05. Multivariate logistic regression analysis was used to determine predictor variables that predict the outcome.

### Ethical consideration

Ethical approval to conduct the study was obtained from the WBUCHS/BMC joint institutional ethic review committee before the commencement of the study. Patients recruited prospectively were required to sign a written informed consent for the study and for HIV testing.

## Results

Out of 1124 patients who presented with peptic ulcer disease (PUD) during the study period, 96 patients underwent emergency laparotomy for perforated peptic ulcers. Of these, 8 patients were excluded from the study due to incomplete data and failure to meet the inclusion criteria. Thus, 84 patients were enrolled giving an average of 17 cases annually and represented 7.5% of cases. Of these, 18 (21.4%) patients were studied retrospectively and the remaining 66 (78.6%) patients were studied prospectively.

### Socio-demographic characteristics

Forty-eight (57.1%) were males and females were 36 (42.9%) with a female ratio of 1.3:1. The patient's age ranged from 12 to 72 years with a median of 32.4 years. The peak incidence was in the 4^th ^decade (31-40 years). The majority of patients, 52 (61.9%) were younger than 40 years. Most of patients, 64 (76.2%) had either primary or no formal education and more than three quarter of them were unemployed.

### Clinical presentation

The duration of symptoms ranged from 1 to 12 days with a mean duration of 6.5 ± 2.3days. The median was 5.8 days. 24 (28.6%) presented within twenty-four hours of onset of symptoms, 25 (29.8%) between 24 and 48 hours and 30 (35.7%) over 48 hours afterwards. The duration of symptoms was not documented in 5 (5.9%) patients. The commonest presenting symptoms were sudden onset of severe epigastric pain in 82 (97.6%), abdominal distention in 64 (76.2%) and vomiting in 31 (36.9%) patients. Abdominal tenderness and classical signs of peritonitis were demonstrable in 74 (88.1%) and 56(66.7%) patients respectively (Table 1).

**Table 1:** Clinical presentation

Clinical presentation	Frequency	Percentage
Severe abdominal pain	82	97.6
Abdominal distention	64	76.2
Vomiting	31	36.9
Nausea	30	35.7
Severe dyspepsia	28	33.3
Constipation	25	29.8
Fever	18	21.4
Shock	28	33.3
Abdominal tenderness	74	88.1
Classical signs of peritonitis	56	66.7

Fifty-eight (69.0%) patients reported no previous history of treatment for peptic ulcer disease. Patients with a previous history of peptic ulcer disease had had symptoms for durations ranging from six months to 14 years and all of them were not on regular anti-ulcer therapy. Three (3.6%) patients presented with re-perforation. Nine (10.7%) patients reported history of recent ingestion of non-steroidal anti-inflammatory drugs (NSAIDS) for joint and back pains. Other risk factors recorded included alcohol consumption and smoking in 72 (85.7%) and 54 (64.3%) patients respectively. Most patients who smoked also took alcohol.

In this study, six (7.1%) patients had associated premorbid illness namely osteoarthritis in 3 patients and hypertension, diabetes mellitus and sickle cell disease in 1 patient each respectively. Eight (9.5%) patients were HIV positive. Of these, 3 (37.5%) patients were known cases on ant-retroviral therapy (ARV) and the remaining 5 (62.5%) patients were newly diagnosed patients. CD4+ count distribution among HIV positive patients ranged from 56 cells/μl to 650 cells/μl with the mean of 236 cells/μl and standard deviation of 86 cells/μl. The median and the mode were 220 cells/μl and 160 cells/μl respectively. A total of two HIV patients (25.0%) had CD4+ count below 200 cells/μl and the remaining 6 patients (75.0%) had CD4+ count of ≥200 cells/μl. Of the eight patients with HIV infection, six (75.0%) patients reported to have risk factors for HIV infection. Of these, alcoholism [Odds Ratio 11.3, 95% C.I. (8.3-16.7), P = 0.021] and multiple sexual partners [Odds Ratio 10.8, 95% C.I. (6.7-14.9), P = 0.000] were found to be independently and significantly associated with increased risk to HIV infection

### Radiological, operative and histopathological findings

Seventy-nine (94.0%) of the patients had plain abdominal and chest radiographs done, with free gas under the diaphragm (pneumoperitonium) demonstrated in 52 (65.8%) of them. All patients in this study underwent laparotomy. The time interval between the beginning of the symptoms of perforation and surgery ranged from12 to140 hours with the median of 72 hours. The majority of patients (76.2%) presented 48 hours or more after the onset of the symptoms of perforation. During operation, all the cases were opened through midline incision and after opening of the peritoneum, there was expulsion of gas in all cases. Most perforations were located on the duodenum {78, 92.9%), whereas in the remaining six (7.1%) patients had their ulcers located on the stomach. The duodenal to gastric ulcers ratio was 12.7: 1. The majority of patients, 82 (97.6%) had single perforation and the remaining 2 (2.4%) patients had both duodenal and gastric perforations. The mean age of the patients with gastric ulcers (56.4 ± 12.5) was significantly higher than that of those with duodenal ulcers (32.8 ± 14.4) (P = 0.002). The median size of the ulcer was 5.4 mm (2-20 mm). Seven (8.3%) of the perforations were found to be sealed. Thirteen (15.5%) of the perforations were of minimal size (≤5 mm) and sixty-four (76.2%) were massive (>10 mm). All perforations were found adhered with omentum and the nature of peritoneal fluid was sero- sanguineous in 34 (40.5%) patients, bilious in 28 (33.3%) patients and purulent in 14 (16.7%) patients. The amount of peritoneal fluid varied from 500 to 1000 mls with a median of 564 mls. The nature of peritoneal fluid was not documented in 8 (9.5%) patients. Histological examination of the biopsy specimens revealed no malignancy. All biopsies were not stained for *Helicobacter pylori.*

### Surgical treatment

The majority of patients, 70 (83.3%) had Graham's omental patch of the perforations with either a pedicled omental patch or a free graft of omentum. Those with sealed perforations had peritoneal lavage with warm saline and mass closure of the abdomen. One patient had truncal vagotomy and Roux-en-Y gastro-jejunostomy in addition to simple closure. One patient who had a large ulcer, which penetrated to the pancreas and caused pyloric obstruction, underwent subtotal gastrectomy.

### Outcome of Treatment

Post-operative complications were recorded in 25 (29.8%) patients. Of these, surgical site infection (48.0%) was the most common post-operative complications (Table 2). The mean age of patients who developed complications was 52.4 ± 16.4 years, whereas the mean age of patients without complications was 32.6 ± 10.2 years. This age difference was statistically significant (P = 0.011). The complication rates for 0, 1, 2 and 3 Boey scores were 8.0%, 12.0%, 20.0% and 60.0%, respectively (P = 0.002, Pearson χ2 test)

**Table 2:** Post-operative complications (N = 25)

Complications	Frequency	Percentage
Surgical site infections	12	48.0
Post-operative pyrexia	9	36.0
Pulmonary infection	7	28.0
Intra-abdominal abscess	5	20.0
Wound dehiscence/burst abdomen	5	20.0
Re-perforation	4	16.0
Septic shock	3	12.0
Enterocutaneous fistula	3	12.0
Peritonitis	3	12.0
Incisional hernia	2	8.0
Cardiopulmonary arrest	2	8.0
Acute renal failure	1	4.0
Paralytic ileus	1	4.0

Table 3 shows predictors of complications according to univariate and multivariate logistic regression analysis. The overall length of hospital stay (LOS) ranged from 1 to 48 days with a median of 14 days. The median LOS for non-survivors was 3 days (range 1-8 days). Patients who developed complications stayed longer in the hospital and this was statistically significant (P = 0.005). In this study, nine patients died giving a mortality rate of 10.7%. The mortality rate increased progressively, with increasing numbers of Boey scores: 0%, 11.1%, 33.3%, and 56.6% for 0, 1, 2, and 3 factors, respectively (P < 0.001, Pearson χ2 test).

**Table 3:** Predictors of complications according to univariate and multivariate logistic regression analysis

Predictor(independent) variable	Complication N (%)	No complication n (%)	Univariate analysis		Multivariate analysis	
			
			**O.R. 95% C.I**.	p-value	**O.R. 95% C.I**.	p-value
**Age (in years)**						
<40	15 (28.8)	37 (71.2)				
≥40	10 (31.2)	22 (68.8)	3.91(0.94-5.23)	0.167	1.23(0.93-2.34)	0.786
**Sex**						
Male	14 (29.2)	36 (70.8)				
Female	11 (30.6)	25 (69.4)	1.87(0.22-4.88)	0.334	3.32(0.45-4.66)	0.937
**Premorbid illness**						
Yes	4 (66.7)	2(33.3)				
No	21(26.9)	57(73.1)	3.54(1.33-5.87)	0.012	5.28(2.39-6.82)	**0.007**
**Previous PUD**						
Yes	7(26.9)	19(73.1)				
No	18(31.0)	40(69.0)	0.21(0.11-1.78)	0.051	1.65(0.32-2.89)	0.786
**NSAIDs use**						
Yes	3(33.3)	6(66.7)				
No	22(29.3)	53(70.7)	1.98(0.99-3.91)	0.923	1.02(0.78-3.90)	0.123
**Alcohol use**						
Yes	22(30.6)	50(69.4)				
No	3(25.0)	9(75.0)	3.05(0.19-2.86)	0.054	0.45(0.22-5.21)	0.321
**Cigarette smoking**						
Yes	17(31.5)	37(68.5)				
No	8(26.7)	22(73.3)	3.11(0.44-5.23)	0.145	3.02(0.99-4.56)	0.334
**Treatment delay**						
< 48	18(90.0)	2(10.0)				
≥ 48	7(14.6)	41(85.4)	1.06(1.01-5.45)	0.021	0.23(0.11-0.95)	**0.003**
**HIV status**						
Positive	6(75.0)	2 (25.0)				
Negative	19(25.0)	57(75.0)	2.87(1.22-4.97)	0.023	1.92(1.31-4.22	**0.001**
**CD4 count**						
**<**200 cells/μl	1 (50.0)	1(50.0)				
≥ 200 cells/μl	1(16.7)	5(83.3)	4.05(3.27-5.01)	0.029	2,94(2.44-6.98)	**0.000**
**Nature of perforation**						
Acute	24(32.4)	50(67.6)				
Chronic	1(10.0)	9(90.0)	4.94(2.84-8.92)	0.009	2.95(1.11-6.98)	**0.018**

Table 4 shows predictors of mortality according to univariate and multivariate logistic regression analysis.

**Table 4:** Predictors of mortality according to univariate and multivariate logistic regression analysis

Predictor (independent) variable	Survivors N (%)	Non-survivors n (%)	Univariate analysis		Multivariate analysis	
			
			O.R. (95% C.I.)	p-value	(O.R. 95% C.I.)	p-value
**Age**						
< 40	51(98.1)	1 (1.9)				
≥40	24(75.0)	8 (25.0)	2.33(1.25-3.42)	0.032	4.61(2.72-7.91)	**0.002**
**Sex**						
Male	42 (87.5)	6 (12.5)				
Female	33 (91.7)	3 (8.3)	1.25 (0.32-3.56)	0.896	2.93 (0.94-3.81)	0.983
**Premorbid illness**						
Yes	2 (33.3)	4 (66.7)				
No	73 (93.6	5 (6.4)	6.21(1.49-7.01)	0.039	3.78(2.98-7.90)	**0.017**
**Previous PUD**						
Yes	23 (88.0)	3(12.0)				
No	52 (89.7)	6 (10.3)	1.75(0.76-4.34)	0.896	3.11(0.98-4.88)	0.345
**HIV status**						
Positive	1(12.5)	7 (87.5)				
Negative	74(97.4)	2 (2.6)	0.56(0.12-0.86)	0.005	1.74(1.21-4.98)	**0.001**
**CD 4+ count**						
< 200 cells/μl	1(50.0)	1 (50.0)				
≥ 200 cells/μl	4(66.7)	2 (33.3)	5.91(2.76-7.99)	0.001	1.65(1,22-7.43)	**0.000**
**Duration of illness**						
<24 hours	23 (92.0)	2 (8.0)				
≥24 hours	48 (87.3)	7 (12.7)	2.32(0.54-6.45)	0.986	0.09(0.02-1.11)	0.315
**Shock on admission (SBP < 90 mmHg)**						
Yes	28 (77.8)	8 (22.2)				
No	47 (87.9)	1(2.1)	7.9(3.98-9.88)	0.022	3,74(2,11-7.76)	**0.005**
**Timing of surgical treatment**						
**<**48 hours	19 (95.0)	1 (5.0)				
≥ 48 hours	56 (87.5)	8 (12.5%)	2.87(2.11-7.21)	0.044	2.91(1.22-6.66)	**0.028**
**Amount of fluid (mls**						
< 200	19 (95.0)	1 (5.0)				
≥200	56(87.5)	8 (12.5)	0.67(0.23-4.65)	0.982	1.61(0.89-2.73)	0.067
**Site of perforation**						
Duodenum	72 (93.4)	5 (6.6)				
Gastric	2 (33.3)	4 (66.7)	5.81(3.33-6.92)	0.012	1.35(1.11-3.86)	**0.018**
**Size of ulcer**						
Sealed	7 (100.0)	0(0)				
<5 mm	12 (92.3)	1(7.7)				
≥5 mm	56 (87.5)	8(12.5)	1.98(0.45-3.82)	0.987	3.13(0.99-4.89)	0.453
**Complications**						
Present	18 (72.0)	7(28.0)				
Absent	57(96.6)	2 (3.4)	1.98(1.54-7.93)	0.005	2.86(2.22-6.45)	**0.011**

### Follow up of patients

Out of 75 survivors, 46 (61.3%) patients were followed up for 6 to 12 months after surgery. Depending upon their symptoms at each visit, patients were classified according to Visick grading system as follows: Visick grade I, 38 (82.6%) patients, Visick grade II, 4 (8.7%) patients, Visick grade III and IV, 2 (4.3%) patients each respectively. One of patients (2.2%) in Visick grade IV presented with re-perforation which necessitated re-operation.

## Discussion

In this review, a total of 84 patients were enrolled over a five year period giving an average of 17 cases annually. This figure is similar to what was reported by Schein *et al *[19]. Mieny *et al *[20] in South Africa reported a low incidence of perforated PUD. These differences reflect differences in the rate of risk factors for perforated peptic ulcer disease from one country to another. The figures in our study may actually be an underestimate and the magnitude of the problem may not be apparent because of high number of patients excluded from this study.

In the present study, perforated peptic ulcer disease were found to be most common in the fourth decade of life and tended to affect more males than females, with a male to female ratio of 1.3:1 which is comparable with other studies in developing countries [3,21-23]. Our demographic profile is in sharp contrast to what is reported in developed countries where the majority of the patients are above 60 years and the incidence is higher in elderly females taking ulcerogenic medications [24]. Male predominance in this age group is attributed to excessive alcohol consumption and smoking among young males which is common in our environment. Alcohol consumption and smoking have been reported to be associated with increased risk for perforated peptic ulcer. Alcohol, as a noxious agent causes gastric mucosal damage, stimulates acid secretion and increases serum gastrin levels [25] and smoking inhibits pancreatic bicarbonate secretion, resulting in increased acidity in the duodenal bulb. It also inhibits the healing of duodenal ulcers [21,26].

The rate of H. pylori infection in patients with perforated peptic ulcers ranges from 50%-80% and *H. pylori *infection, as a risk factor for perforated PUD, appears to be more relevant in younger patients. This is in contrast to elderly patients, where NSAIDs may play a more significant etiologic role [27]. Determination of *Helicobacter Pylori *was not performed in our study due to lack of reagents.

Use of NSAID is an important cause of perforated peptic ulcer in the West. In our series, NSAID use as an offending cause could be attributable in only 10.7% patients. NSAID inhibit prostaglandin synthesis so further reducing gastric mucosal blood flow [27].

In agreement with other studies [3,24], more than sixty percent of patients had no past history suggestive of peptic ulcer disease and those with a known history of PUD were not on regular treatment. This is in sharp contrast to Nuhu *et al *in Nigeria who reported that 71% of cases had previous history of peptic ulcer disease [21]. It has been reported that in many developing countries, the diagnosis of PUD is first made in many instances after perforation [28]. The present study confirms this observation because more than sixty percent of the patients with perforation were not diagnosed previously as cases of PUD and therefore were not on treatment. Patients with no previous diagnosis of peptic ulcer have a higher risk of PUD perforation than patients with a known history of ulcer disease. This may be because preventative measures are more likely to have been taken in patients with a known history of ulcer. Furthermore, these patients are perhaps more likely to seek treatment earlier.

In this study, most of patients had either primary or no formal education and more than three quarter of them were unemployed. Similar occupational pattern was reported by others [21,22]. This observation has an implication on accessibility to health care facilities and awareness of the disease.

It has been reported that the interval between perforation and initiation of treatment is a better predictor of outcome. In the present study most of patients presented late more than 24 hours from the start of symptoms. This is in agreement with other studies in most developing countries [3,21-23,28]. Late presentation in our study may be attributed to lack of accessibility to health care facilities and lack of awareness of the disease. Hospital treatment is expensive and the patients may seek care only when the pain is unbearable. Patients may take medications in the pre-hospital period with hope that the symptom will abate. It is also possible that some clinicians managing the patients initially may not have considered perforation as a possible diagnosis.

More than 90% of our patients had classical presentation with sudden onset of sharp epigastric pain, as most of the studied patients were young aged in contradistinction to elderly patients in whom silent perforations usually occur [3,29].

As reported in other studies [5,9,30], associated premorbid illness was documented in 7.1% of cases. Associated premorbid illnesses have been reported to influence the outcome of patients with perforated peptic ulcers [5]. In the present study, associated premorbid illness predicted the outcome of patients with perforated peptic ulcers.

The prevalence of HIV infection among patients with perforated PUD in the present study was 9.5% that is higher than 6.5% [31] in the general population in Tanzania. This difference was statistically significant (P < 0.001). The high prevalence of HIV infection in our patients may be attributed to high percentage of the risk factors for HIV infection reported in the present study population. The overall HIV seroprevalence in our study may actually be an underestimate and the magnitude of the problem may not be apparent because many cases (8 patients) were excluded from the study due to failure to meet the inclusion criteria. We could not find any literature regarding the effect of HIV infection on the perforation rate and outcome in patient with perforated PUD. This calls for a need to research on this observation. In this study, HIV infection was found to be associated with high perforation rate and poor postoperative outcome. This observation calls for routine HIV screening in patients suspected to have perforated PUD.

In agreement with other studies [3,4,21,22,32], the diagnosis of perforated PUD in this study was made from history and identification of free air under the diaphragm in plain abdominal and chest radiographs, and the diagnosis was confirmed at laparotomy. The value of the radiological investigation has been compared with other writers and with current radiological techniques; 80-90% of cases are correctly diagnosed [4,33]. In case of perforated PUD ulcer, free intraperitoneal gas is less likely to be seen if the time interval between the perforation and radiological examination in short [4]. Recently, Computerized tomography (CT) scans with oral contrast are now considered the reliable method of detecting small pneumoperitonium before surgery and the gold standard for the diagnosis of a perforation [34,35]. Abdominal ultrasonography has also been found to be superior to plan radiographs in the diagnosis of free intra-peritoneal air [35]. None of these imaging studies were used in the diagnosis of free intra-peritoneal air in our study. We relied on plain radiographs of the abdominal/chest to establish the diagnosis of free intra-peritoneal air which was demonstrated in 65.8% of cases. We could not establish, in our study, the reason for the low detection rate of free air under the diaphragm.

In our study, duodenal ulcer perforation was the most common type of perforation with a duodenal to gastric ulcer ratio of 12.7:1. This is comparable to a study in Kenya which reported a duodenal to gastric ulcer ratio of 11.5:1 [32]. A high duodenal to gastric ulcer ratio of 25:1 was reported in Sudan [36]. A study in Ghana reported high incidence of gastric ulcer perforations than duodenal ulcer perforation [37]. Low duodenal to gastric ulcer ratios of 3:1 to 4:1 have been reported from the western world [32,37]. Gastric ulcer is considered a rare disease in Africa being 6-30 times less common than duodenal ulcers [37,38]. There was no obvious explanation to account for these duodenal to gastric ulcer ratio differences.

In this study, Graham's omental patch of the perforations with either a pedicled omental patch or a free graft of omentum was the operation of choice in our centre. Similar surgical treatment pattern was reported in other studies [3,4,21,22]. This is a rapid, easy and life-serving surgical procedure that has been shown to be effective with acceptable mortality and morbidity [22,39]. Although this procedure has been associated with ulcer recurrence rates of up to 40% in some series, Graham's omental patch of PUD perforations remains a surgical procedure of choice in most centres and to avoid recurrence the procedure should be followed by eradication of *H. pylori *[22,40]. Simple closure of perforation with omental patch and the use of proton pump inhibitors have changed the traditional definitive peptic ulcer surgery of truncal vagotomy and drainage procedures [41]. Definitive surgery is indicated only for those who are reasonably fit and presented early to the hospital for surgery [22]. Definitive peptic ulcer surgery increases operative time, exposes the patient to prolonged anaesthesia and also increases the risk of postoperative complications. This is especially true in developing countries including Africa where patients often present late with severe generalized peritonitis [23]. In the present study, only one patient who presented early with stable haemodynamic state underwent definitive peptic ulcer surgery of truncal vagotomy and drainage. Recently, laparoscopic repair of perforated peptic ulcer has also been reported, [42] and this is believed to help reduce postoperative morbidity and mortality [43].

The laparoscopic technique in closure of perforated peptic ulcers is being practiced in several centres in developed countries [42,43], it has not yet been tried in any of our hospitals in this country.

Overall complications rate in this series was 29.8% which is comparable to what was reported by others [4,44]. High complications rate was reported by Montalvo-Javé *et al *[6]. This difference in complication rates can be explained by differences in antibiotic coverage, meticulous preoperative care and proper resuscitation of the patients before operation, improved anesthesia and somewhat better hospital environment. In keeping with other studies [21,22,39], surgical site infection was the most common complication. High rate of surgical site infection in the present study may be attributed to contamination of the laparotomy wound during the surgical procedure.

Perforated peptic ulcer is a serious condition with an overall reported mortality of 5%-25%, rising to as high as 50% with age [5-7,9,11,44]. In this study mortality rate was high in patients who had age ≥ 40 years, delayed presentation (>24 hrs), shock at admission (systolic BP < 90 mmHg), HIV positivity, low CD4 count (< 200 cells/μl) and concomitant diseases. Also gastric ulcers were associated with an increased mortality risk. Boey's score, which is a score based on scoring factors as shock on admission, confounding medical illness, and prolonged perforation, has been found to be a useful tool in predicting outcome [11]. In this study, Boey score was a good predictor of both mortality and postoperative complication and therefore should be used in our setting as a tool for predicting outcome in patients with perforated peptic ulcers.

Since tests for detecting *H. Pylori *was not possible in our patients due to logistic problems, we did not take this into consideration in our discussion. However the use of the 'triple regime' produced excellent results in 82.6% of our patients which is comparable to the results from recent studies [3. 4, 21, 22, 45] which have successfully used simple closure followed by eradication of *H-Pylori *as a treatment for perforated peptic ulcer. This is in contrast to the earlier studies [46,47] which reported emergency definitive surgery as a means to prevent recurrence and re-operation rates. These findings are extremely important for developing countries like Tanzania where delay in presentation often prevents any attempt at definitive surgery.

Before generalizing the results of our study several important issues need to be addressed. First, since all the subjects in the present study underwent pen repair, results from this study may not fully represent those after laparoscopic repair. Second, we did not study the association of *H. pylori *with the postoperative outcomes because of lack of necessary facilities at the study center. Third, data obtained retrospectively and failure to detect HIV infection during window period may have underestimated the prevalence of HIV infection. Fourth, since our duration of postoperative follow up was relatively short, we could not estimate the long term effect of Graham's omental patch.

## Conclusion

Perforation of peptic ulcer remains a frequent clinical problem in our environment predominantly affecting young males not known to suffer from PUD. Simple closure with omental patch followed by *Helicobacter pylori *eradication was effective with excellent results in majority of cases despite patients' late presentation in our center.

## Competing interests

The authors declare that they have no competing interests. The study had no external funding. Operational costs were met by authors

## Authors' contributions

PLC - study design, literature search, data analysis, manuscript writing & editing and submission of the manuscript, JBM, MK, MDM, HMJ, RK, ABC participated in data analysis, manuscript writing & editing and JMG- supervised and coordinated the manuscript writing & editing. All the authors read and approved the final manuscript.
